# Lumbar medial branch block progression to radiofrequency neurotomy: A retrospective audit of clinical practice

**DOI:** 10.1016/j.inpm.2021.100009

**Published:** 2022-01-06

**Authors:** David Sherwood, Evan Berlin, Benjamin Gill, Adam Epps, James Gardner, Byron Schneider

**Affiliations:** a Department of Orthopedics, University Health Lakewood Medical Center, Kansas City, MO, USA; b Department of Physical Medicine and Rehabilitation, Vanderbilt University Medical Center, Nashville, TN, USA; c Department of Physical Medicine and Rehabilitation, University of Missouri, Columbia, MO, USA

**Keywords:** Spine, Neurotomy, Ablation, Radiofrequency, Pain Management, Low Back Pain, Facet Joint, Z-joint, Zygapophysial Joint, Zygapophyseal joint, Chronic pain, Nonoperative

## Abstract

**Introduction:**

Chronic axial low back pain due to zygapophysial joint arthropathy is best diagnosed via lumbar medial branch block (MBB). However, the paradigm by which MBB is used to select patients for lumbar radiofrequency neurotomy (RFN) is contested. Dual diagnostic lumbar MBB with a minimum of ≥80% pain relief to diagnose lumbar zygapophysial joint pain are accepted by some Medicare Local Coverage Determination (LCD) as the method for selecting patients for RFN for the management of lumbar zygapophysial joint pain. However, some argue that dual diagnostic MBB and the ≥80% pain relief threshold lack utility in clinical practice, given that those that progress from MBB1 to MBB2 will then flow from MBB2 to RFN without fail.

**Study:**

Pragmatic retrospective clinical audit.

**Objective:**

Does clinical practice of dual diagnostic lumbar MBBs and an ≥80% pain improvement diagnostic threshold reduce patient eligibility for RFN after both MBB1 and MBB2?

**Results:**

Using dual diagnostic lumbar MBBs and an ≥80% pain improvement diagnostic threshold, 90/167 (54%, 95% CI 46–61%) patients successfully progressed from MBB1 to MBB2. Of those 90 patients, 66 patients (73%, 95% CI 64–82%) successfully progressed from MBB2 to RFN. Both MBB1 and MBB2 impacted the eligibility of the progression of 77/167 (46%, 95% CI 39–54%) patients and 24/90 patients (27%, 95% CI 18–36%), respectively. An additional sub-cohort analysis which included all the patients from the ≥80% pain relief cohort, and those who progressed at the discretion of the providers with 50–79% relief revealed that 124/167 patients (74%, 95% CI 68–81%) successfully progressed from MBB1 to MBB2. Of those 124 patients, 99 patients (80%, 95% CI 73–87%) progressed from MBB2 to RFN. In this laxer criteria cohort, MBB1 and MBB2 impacted the eligibility of the progression of 43/167 patients (26%, 95% CI 19–32%) and 25/124 patients (20%, 95% CI 13–27%), respectively.

**Conclusion:**

MBB1 and MBB2 both filtered patients from progression to lumbar RFN using dual MBBs with an ≥80% pain relief criteria. It also held true when using a more relaxed pain relief selection criterion as well. Dual MBB's and ≥80% pain improvement criteria as a selection paradigm led to half as many lumbar RFNs being performed when compared to a single MBB and ≥80% pain improvement criteria. In theory, a more rigid selection paradigm treats less patients but exposes fewer to unnecessary RFNs while a laxer selection paradigm treats more patients but exposes more to unnecessary RFNs.

## 1 Introduction

Acute low back pain is common and has a favorable natural course [1,2]. However, chronic axial low back pain (CALBP), defined as pain greater than 3 months without the presence of radicular or myelopathic symptoms, is less common but among the most costly and debilitating diagnoses [[3], [4], [5], [6]]. There are many phenotypes of CALBP, which can be differentiated by thoughtful evaluation and intervention [[7], [8], [9], [10]]. Based on the cohort examined and criteria for selection, the zygapophysial joint, also known colloquially as the z-joint or facet joint, is implicated in 5–50% of CALBP cases [[11], [12], [13], [14], [15], [16], [17], [18], [19], [20]]. The most rigorous diagnostic selection methodology revealed a 15% prevalence [21]. Radiofrequency neurotomy (RFN) of the associated lumbar medial branches is a validated and efficacious intervention to treat this population [13,18,[22], [23], [24]]. No physical exam maneuvers, historical features, or imaging studies can independently diagnose CALBP of zygapophysial joint origin [13,17,25,26]. The diagnosis of zygapophysial joint pain is best achieved by medial branch block (MBB) [12,13,15,16,19,[26], [27], [28], [29], [30]]. [][12], [13], [15], [16], [19], [26], [27], [28], [29], [30][].

The paradigm through which MBB is used to diagnose zygapophysial joint pain is contested given the false positive rate for a single lumbar MBB is within the range of 15–45% [11,12,18,19,23,[25], [26], [27],[30], [31], [32]]. Some practitioners argue for a single MBB, some argue for dual MBBs, and some argue for a no MBB protocol for the diagnosis and treatment of CALBP of zygapophysial etiology. Moreover, there are differing opinions regarding which threshold of pain relief percentage denotes success with the MBB. The specifics of these debates are important but have been discussed previously and are beyond the scope of the present objective [15,23,25,27,29,30,[33], [34], [35]]. [][15], [23], [25], [27], [29], [30], [33], [34], [35][].

Dual MBBs with an 80% or greater pain relief cut off to diagnose zygapophysial joint pain have been accepted by the Spine Intervention Society (SIS) and some Medicare LCDs as the most appropriate method for diagnosing lumbar zygapophysial joint pain [36,37]. Some published consensus guidelines debate the merits of this selection criteria [30]. The argument against the dual block paradigm and the 80% or greater pain relief cut off is that a less stringent selection criteria may treat more patients and save healthcare dollars but potentially at the expense of more treatments upon patients that were false positives. Moreover, there are some who argue anecdotally that all patients who matriculate from the first MBB to the second MBB graduate to RFN, thus questioning the value of MBB2 in their practice setting.

The Spine Intervention Society endorses a dual block paradigm using an 80% or greater pain relief cut off given a preponderance of research demonstrating the highest positive outcome rates when patients are enrolled via this paradigm [36]. In 2020, Schneider et al. produced a systematic review which stratified qualifying data to demonstrate that using this patient selection paradigm, physicians may expect a 50–60% chance of patients achieving 50% relief, a 50% chance of achieving 80% relief, and a 25% chance of achieving complete relief along with improvements in function and decreased use of analgesics [23]. By advocating for these selection criteria, the Spine Intervention Society has made an endorsement towards “precision medicine” in the field of Interventional Pain Management.

The 2020 Multisociety International Working Group Consensus practice guidelines on lumbar facet joint pain recommend a single block using 50% or greater pain relief as the cut off [30]. Those consensus guidelines recognize that a no block paradigm likely benefits the most patients at the expense of treating some who may not benefit, while a dual block paradigm likely produces the highest rates of successful RFN outcomes but at the expense of withholding treatment for some who may benefit [30]. The consensus guidelines examined this statistical Rorschach test, and thus recommended a reasonable middle ground single block paradigm. The guidelines state that this decision allows physicians to practice “personalized medicine” tailored to their patient's needs, such that there exist contexts in which either a no block, single block, or dual block paradigm are most appropriate [30].

By not endorsing a dual block paradigm, it can be reasonably inferred that the authors of the consensus guidelines feel that the second medial branch block (MBB2) does not typically confer utility in clinical practice. Our research attempts to add further data to the conversation on the utility of MBB2 in clinical practice.

There have been prior seminal and valued studies which have assessed the significance of differing block paradigms as it relates to RFN outcomes, prognostic ability of differing pain-relief cut offs for RFN outcomes, and the financial cost of differing block paradigms [14,35,[41], [42], [43]]. Nested within those studies exist data, which is like our own, but importantly not identical. Our data is uniquely positioned as to address if prospective trial data regarding the utility of MBB1 and MBB2 to both prevent matriculation to RFN at some degree is manifested in clinical practice.

No prior studies have retrospectively audited clinical practice using the dual block paradigm and 80% or greater pain relief criteria to the dedicated assessment of whether this methodology reduce the number of patients who flow from MBB to RFN at both MBB1 and MBB2.

Our study is a retrospective review protocol study that set out to challenge this suspicion, does clinical practice using dual diagnostic lumbar MBBs and an 80% or greater pain relief reduce the number of patients who matriculate from MBB1 to MBB2 to RFN?

## 2 Methods

Institutional Review Board (IRB) approval was obtained. A database was retrospectively established with all recipients who underwent lumbar medial branch blocks based on Current Procedural Terminology (CPT) codes within a single department at a large academic institution between January 1st, 2019 and December 31st, 2019. Four fellowship-trained physiatrists performed all procedures in this study.

### 2.1 Procedural technique

The procedural technique for each provider noted in their associated procedure notes was consistent with the guidelines established by the Spine Intervention Society for MBB of the lumbar spine. [36] No patient was offered MBB1 before completing 4–6 weeks of conservative care. No patient received sedation. While data was not collected on local anesthetic usage, 2/4 of the physicians reviewed never use local anesthetic for this procedure while the remaining 2/4 physicians rarely use local anesthetic for this procedure. When local anesthetic was used, it was 2% lidocaine injected as a subdermal wheel using roughly 0.5 ​mL's. At the provider's discretion, 2% lidocaine or 0.5% bupivacaine were used for either MBB1 or MBB2. The same anesthetic was not used for both MBB1 and MBB2. No provider used steroids. No more than 0.5 ​cc per unilateral level was used. To reduce false negatives, contrast was always used before the anesthetic was injected to assess for vascular uptake and appropriate localization of the injectate.

### 2.2 Pain relief criteria

A minimum of 80% or greater pain relief determined a diagnostic MBB per our retrospective protocol. However, as this was a pragmatic retrospective review of clinical practice, each provider could matriculate patients with a ≥50% pain improvement threshold based on clinical judgement, as this was the insurance standard during the study time frame. Patients diagnosed via this method were identified separate from the studied cohort. Ultimately, the preferred tool for defining pain relief and thus matriculation to RFN was at the discretion of the provider. Those measurement tools included: (1) pre and post injection NRS or VAS data, (2) patient pain diaries of NRS and percentage relief scoring, and (3) post-injection follow-up visits where NRS or VAS scores were collected by patient recall.

### 2.3 Inclusion and exclusion criteria

#### 2.3.1 Inclusion criteria

1. First lumbar medial branch block was performed between January 1st, 2019 and December 31st, 2019.
2. The procedure only targeted the lumbar medial branches or the L5 dorsal ramus.

#### 2.3.2 Exclusion criteria

1. Patients received a lumbar medial branch block or neurotomy before January 1st, 2019 to the same level and laterality.
2. The procedure targeted either the cervical or thoracic medial branches, or the sacral lateral branches.
3. The patient did not undergo medial branch block, rather a procedure with the same CPT code (e.g., facet injection or costovertebral joint injection)

The medical records of patients selected with the above criteria were individually reviewed by a team of five physicians. The researchers collected data on the patient's demographics (i.e., age, sex), duration of pain, target nerve levels, pre-procedure pain, post-procedure pain, pain relief duration of the MBBs, if patient proceeded to the second MBB, the rationale for failure to matriculate if documented, and if the patient proceeded to RFN.

If the patient did not follow-up after the first or second medial branch block, regardless of immediate post-procedure pain score, it was considered a failure to matriculate. All data were stored in password-secured documents. Data shared between researchers was sent via encrypted emails with no patient identifiable information, in accordance with the Health Insurance Portability and Privacy Act.

## 3 Results

A total of 167 patients underwent lumbar MBB and fulfilled the inclusion and exclusion criteria. The mean duration of pain was 49.3 months. The mean numeric rating score (NRS) pre-MBB1 was 6.0. The mean NRS pre-MBB2 was 5.3. The mean time between MBB1 and MBB2 was 26 days. There were 54 unilateral and 113 bilateral patients. The L1-2, 2–3 joints were targeted in 6 patients (3.6%), the L2-3 joint was targeted in 2 patients (1.2%), the L2-3, L3-4 joints were targeted in 6 patients (3.6%), the L2-3, L3-4, L4-5 joints were targeted in 3 patients (1.8%), the L3-4 joint was targeted in 4 patients (2.4%), and the L4-5, 5–1 joints were targeted in the remaining 146 patients (87.4%). Aside from insufficient improvement in reported pain, other reasons were identified in the medical records for lack of progression to RFN. See Table 1.

**Table 1:** List of alternative reasons collected for lack of progression.

Reason	MBB1	MBB2
Declined Further Treatment	1	2
Lost to Follow Up	2	4
Sustained Pain Relief	3	2
Excessive Discomfort	1	0
Insurance Denial	0	1

### 3.1 ≥ ​80% pain relief in both MBB1 and MBB2 cohort

**Fig. 1:**
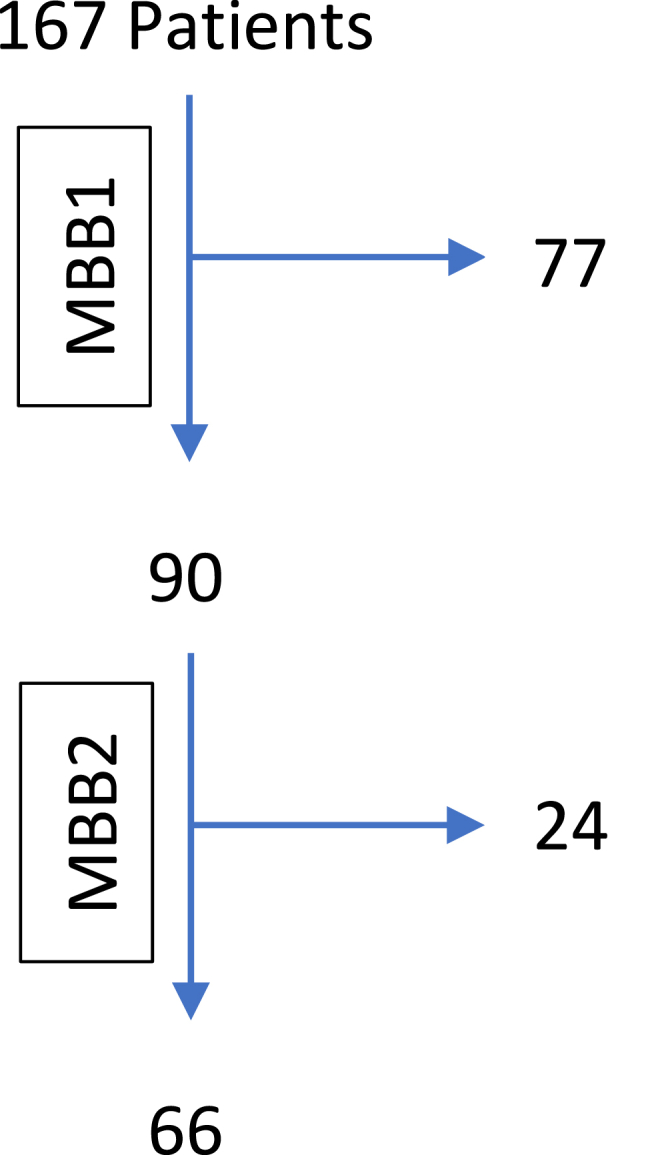
Flow of patients from the first medial branch block to the second medial branch block to radiofrequency neurotomy in the ≥80% pain relief cohort.

Based on a threshold of 80% pain improvement, 90/167 patients (54%, 95% CI 46–61%) progressed from MBB1 to MBB2 after ≥80% pain improvement. Of those 90 patients, 66 patients (73%, 95% CI 64–82%) also progressed from MBB2 to RFN. Ultimately, 66/167 (40%, 95% CI 32–47%) patients who were initially suspected to have zygapophysial joint pain progressed to RFN. The application of the ≥80% relief screening threshold in both MBB1 and MBB2 impacted the RFN eligibility of 77/167 patients (46%, 95% CI 39–54%) and 24/90 patients (27%, 95% CI 18–36%), respectively. See Fig. 1.

### 3.2 Clinical practice, ≥50% pain relief in both MBB1 and MBB2 cohort

**Fig. 2:**
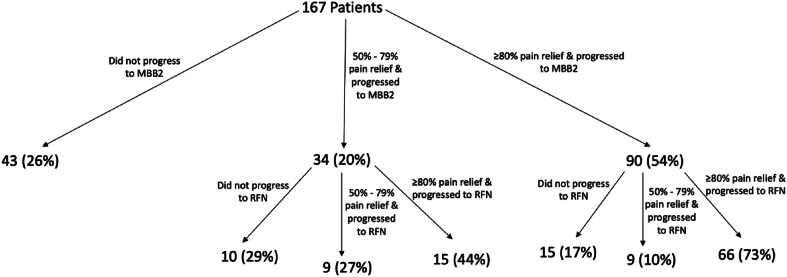
Flow of patients from the first medial branch block to the second medial branch block to radiofrequency neurotomy in the clinical practice cohort.

This cohort includes all the patients from the ≥80% pain relief in both MBB1 and MBB2 cohort, and those who progressed at the discretion of the providers with 50–79% relief. In total, 124/167 patients (74%, 95% CI 68–81%) successfully progressed from MBB1 to MBB2. Of those 124 patients, 99 patients (80%, 95% CI 73–87%) progressed from MBB2 to RFN. Ultimately, 99/167 patients (59%, 95% CI 52–67%) who were initially suspected to have potential CALBP due to zygapophysial joint pain progressed to RFN. In our clinical practice audit, MBB1 and MBB2 impacted the eligibility of the progression of 43/167 patients (26%, 95% CI 19–32%) and 25/124 patients (20%, 95% CI 13–27%), respectively. See Fig. 2.

Of note, 9/167 (5%, 95% CI 2–9%) patients had ≥80% pain relief after MBB1 but 50%–79% pain relief after MBB2 yet still advanced to RFN. Similarly, 15/167 (9%, 95% CI 5–13%) patients had 50%–79% pain relief after MBB1 and ≥80% pain relief after MBB2 yet still advanced to RFN.

## 4 Discussion

In this retrospective review from January 1st, 2019, to December 31st, 2019, of practice habits reflective of SIS and some Medicare LCD guidelines of dual diagnostic lumbar MBBs with an ≥80% pain relief criteria, both MBB1 and MBB2 served to filter patients from progression to RFN. Moreover, this held true using either ≥80% pain relief or the less rigorous clinical practice cohort. In both selection habits, patients were filtered from RFN progression at both MBB1 and MBB2.

Chronic axial low back pain is admittedly challenging to treat. Despite improved diagnostic specificity to individual pathologies, there remains a lack of robustly validated interventions to manage each concern [7,8,[38], [39],^40^]. Inconsistent efficacy with interventions is at least partially due to differing patient selection methods and subsequent heterogeneous data sets. As demonstrated in broad analysis, outcomes for RFN tend to worsen with less selective criteria [23]. Enhanced identification of successful interventions will perhaps occur with consistent and prolonged application of dual diagnostic MBB with an ≥80% pain relief threshold. Furthermore, use of single-institution registries and large insurance-based datasets may also be leveraged to assess pragmatic outcomes of procedures such as RFN. Clinical over-utilization of this procedure in patients without lumbar zygapophyseal pain carries a theoretical risk of poorer outcomes, which may ultimately restrict access of the procedure to the entire population.

The lack of RFN outcome data is addressed in *Limitations*. However, despite no RFN outcome data, some inferences may be made. If our practice had utilized a single block paradigm and 50% pain relief cut off as endorsed by the Multisociety Consensus Practice Guidelines, 124 RFN's would have been carried out compared to the 66 using the dual block paradigm and 80% pain relief cut off as endorsed by the Spine Intervention Society [30,36]. In our clinical audit, the Consensus guidelines exposed an additional 66 patients to RFN, or a 200% increase over the SIS endorsed guidelines.

Our research perhaps corroborates that using a dual block paradigm and 80% or greater pain relief cutoff in the clinical setting is needed to replicate the patient selection in research which shows the highest RFN outcomes. Moreover, by relaxing that rigor, we should expect outcome rates to worsen, yet we may treat more patients. Cohen et al. (2010) masterfully compared multiple selection paradigms to demonstrate that a dual block paradigm was the most precise when selecting patients for successful RFN, albeit at the expense of those who were filtered out by either block who may have benefited [35]. While our study did not aim to replicate that of Cohen, our study did pragmatically evaluate the utilization of RFN in clinical practice when a two-block paradigm is used, 66 RFNs, and be extension how many RFNs would have occurred if only a single block paradigm and 50% pain relief cutoff had been applied, 124 RFNs [30].

### 4.1 Clinical practice, ≥50% pain relief in both MBB1 and MBB2 cohort

The physicians involved in this study practice at a large, research-oriented academic institution. Overall, a firm criterion of ≥80% reported pain improvement was favored for progression to RFN. However, some patients progressed with greater than 50% relief but without fulfilling the selection criteria currently endorsed by SIS and some Medicare LCD guidelines. Specifically, 34/167 (20%, 95% CI 14–26%) advanced from MBB1 to MBB2 and 33/124 (27%, 95% CI 19–34%) advanced from MBB2 to RFN without demonstrating 80% relief at MBB1 and/or MBB2.

In 2010, Cohen et al. reported using a dual block ≥50% pain relief criterion to demonstrate 29/50 (58%) progression from MBB1 to MBB2, and 14/26 (54%) progression from MBB2 to RFN [35]. In our study, 124/167 (74%) progressed from MBB1 to MBB2 and 99/124 patients (80%) progressed from MBB2 to RFN when using a dual block ≥50% pain relief criterion. These differences may be explained by differing patient populations and differing patient selection for both the procedure itself and advancement.

In this cohort, there was a common scenario in which a patient's pain scores would reduce from a 7 to a 2, or a 4 to a 1. In both scenarios, a passive observer might deem these results successful. However, by use of the ≥80% pain relief criteria, both the 7 to 2 and 4 to a 1 patient's would not have been deemed successful enough to further matriculate to the next stage as their pain relief is statistically 71% and 75%, respectively. Moreover, certain patients had a MBB carried out with the expressed understanding that complete pain relief was not a realistic expectation given underlying medical comorbidities which provided an inherent level of pain or alternative spinal pain generators which may not have been amenable to treatment. Thus, in these patients the ≥80% pain relief criteria were felt to be an unfair hurdle to define success.

In this practice of physicians, the clinical decision to progress when the results were between 50 and 79% resulted in some additional procedures but were not universally grounds for progression. We would argue that by allowing physicians leeway with regards to less rigid matriculation such that they may use clinical judgement to assess RFN success potential would remedy both the previously discussed issues we encountered. By allowing physicians to exercise their judgement of the patients in front of them, then perhaps the previously described incidents which might restrict care could be limited.

### 4.2 Limitations

Several limitations were identified within the course of this study. Regarding heterogeneity, the charts reviewed reflect the practice habits of four interventionalists. The ultimate selection criteria for medial branch block candidacy were at the discretion of each provider. Furthermore, the methods of pain threshold determination and reporting were non-standardized between physicians. Therefore, any associated findings lack generalizability given presumed differences in patient selection or referral patterns.

As noted above, there are many reasons why a patient may not matriculate to RFN after MBB. While primarily due to inadequate pain relief based on diagnostic protocols, the figures in this study do not accommodate other reasons for cessation of progression. The lack of discrimination introduces a “best case” analysis bias in favor of the dual block paradigm. However, the aim of this analysis was an overall audit of clinical practice regarding the use of staged blocks as diagnostic filters rather than an elaboration of factors preventing RFN.

Many readers may seek outcome data related to these procedures. However, due to a lack of consistent post-RFN follow-up and data collection, outcome data would be potentially unreliable and misleading. Ultimately, this study was not designed to evaluate the outcomes of RFN.

## 5 Conclusion

This retrospective review of the practice habits of four spine providers at large tertiary academic spine center demonstrated filtration of patient progression to RFN from both MBB1 and MBB2 with use of ≥80% pain improvement criteria. The ≥80% threshold impacted RFN eligibility of 77/167 patients (46%, 95% CI 39–54%) following MBB1, and 24/90 patients (27%, 95% CI 18–36%) following MBB2. Impaired matriculation was also seen with less rigorous selection criteria. Dual MBB's and ≥80% pain improvement criteria as a selection paradigm led to half as many lumbar RFNs being performed when compared to a single MBB and ≥80% pain improvement criteria. In theory, a more rigid selection paradigm treats less patients but exposes fewer to unnecessary RFNs while a laxer selection paradigm treats more patients but exposes more to unnecessary RFNs.

## Conflicts of interest

The authors declare no conflicts of interest.

## Funding

The authors have no sources of funding to declare for this manuscript.
